# Leveraging single‐cell sequencing to unravel intratumour heterogeneity and tumour evolution in human cancers

**DOI:** 10.1002/path.5914

**Published:** 2022-05-23

**Authors:** Amy L Bowes, Maxime Tarabichi, Nischalan Pillay, Peter Van Loo

**Affiliations:** ^1^ Cancer Genomics Group The Francis Crick Institute London UK; ^2^ Sarcoma Biology and Genomics Group UCL Cancer Institute London UK; ^3^ Institute for Interdisciplinary Research Université Libre de Bruxelles Brussels Belgium; ^4^ Department of Histopathology The Royal National Orthopaedic Hospital NHS Trust London UK; ^5^ Department of Genetics The University of Texas MD Anderson Cancer Centre Houston TX USA; ^6^ Department of Genomic Medicine The University of Texas MD Anderson Cancer Centre Houston TX USA

**Keywords:** clone, subclone, intratumour heterogeneity (ITH), tumour evolution, copy number aberrations (CNAs), tumour phylogeny, single‐cell DNA sequencing

## Abstract

Intratumour heterogeneity (ITH) and tumour evolution are well‐documented phenomena in human cancers. While the advent of next‐generation sequencing technologies has facilitated the large‐scale capture of genomic data, the field of single‐cell genomics is nascent but rapidly advancing and generating many new insights into the complex molecular mechanisms of tumour biology. In this review, we provide an overview of current single‐cell DNA sequencing technologies, exploring how recent methodological advancements have enumerated new insights into ITH and tumour evolution. Areas highlighted include the potential power of single‐cell genome sequencing studies to explore evolutionary dynamics contributing to tumourigenesis through to progression, metastasis, and therapy resistance. We also explore the use of *in situ* sequencing technologies to study ITH in a spatial context, as well as examining the use of single‐cell genomics to perform lineage tracing in both normal and malignant tissues. Finally, we consider the use of multimodal single‐cell sequencing technologies. Taken together, it is hoped that these many facets of single‐cell genome sequencing will improve our understanding of tumourigenesis, progression, and lethality in cancer, leading to the development of novel therapies. © 2022 The Authors. *The Journal of Pathology* published by John Wiley & Sons Ltd on behalf of The Pathological Society of Great Britain and Ireland.

## Introduction

In 1890, the German pathologist David Paul Hansemann was one of the first to observe asymmetrical nuclear divisions and failed chromosomal segregation in epithelial cancers [1, 2]. Subsequently, Hansemann coined the term ‘anaplasia’ to describe the reversion of differentiation and ensuing morphological nuclear changes associated with atypical mitoses in cancer cells [1, 2]. Contemporaneously, further debate on the origin of tumours was fuelled by the husband‐and‐wife team of Theordore and Marcella Boveri, who elegantly demonstrated the relevance of chromosome mis‐segregation in heredity and cancer development [2, 3]. The Boveri's proposed that human cancers originate from a single cell with an abnormality of its chromosomal constitution, which is passed onto all descendant cells due to rapid cell proliferation [2, 3]. Thus, the chromosome theory of cancer was born, which has been corroborated and expanded by a wealth of succeeding molecular studies investigating the genetic basis of tumour development and progression [4, 5, 6].

**Figure 1:**
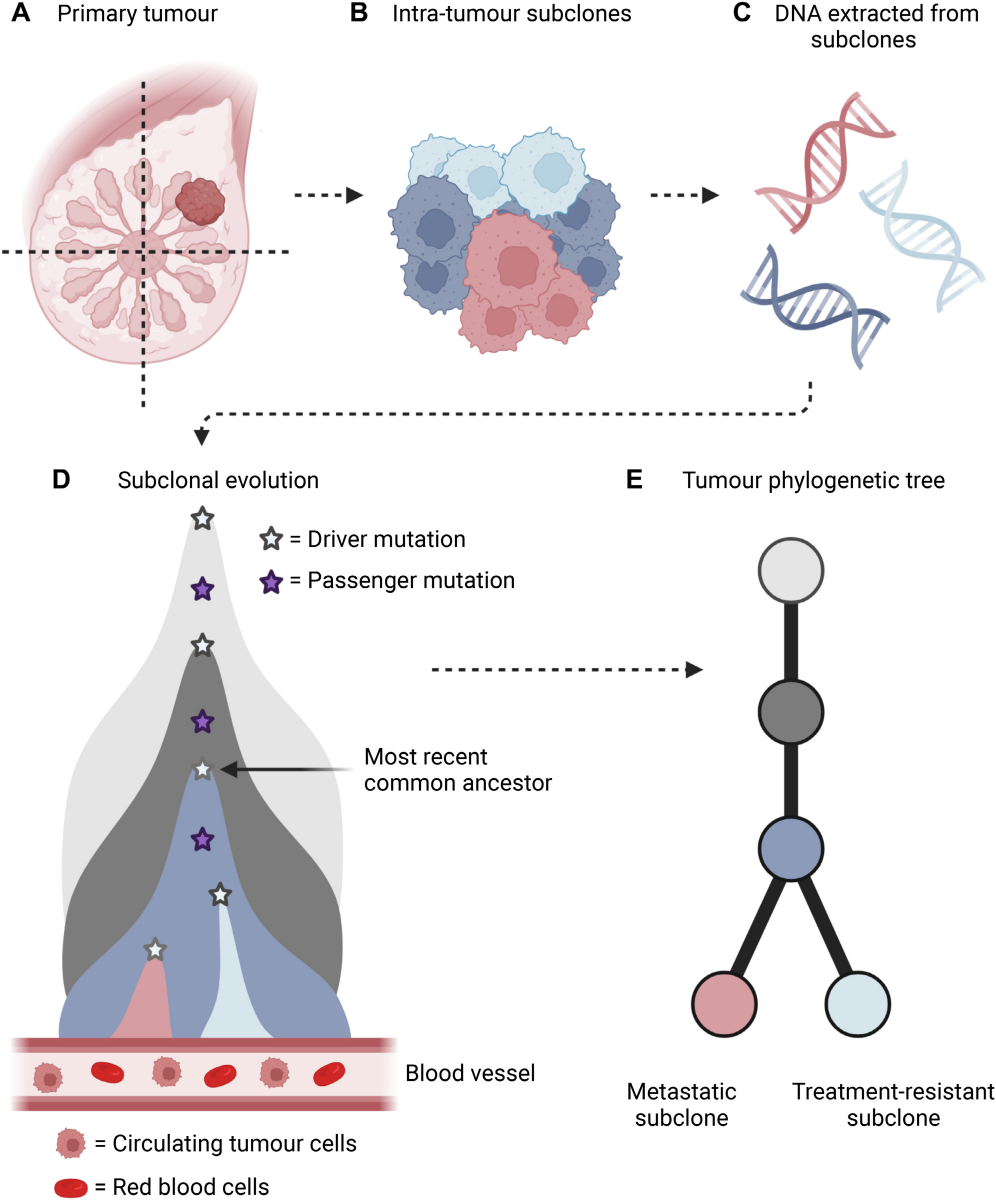
The subclonal architecture of human cancers. (A,B) A primary tumour may contain multiple subclones. (C) Subsequently, the DNA extracted from the tumour will contain intermixed signals from all subclones. (D) Following genetic sequencing, somatic mutations are called, including driver and passenger mutations. Somatic mutations belonging to the MRCA can be identified in all tumour subclones reflecting the last complete clonal sweep. The emergence of new mutations may give rise to additional tumour subclones with further phenotypic features. (E) Somatic mutations can also be leveraged to infer tumour phylogenies. Figure prepared with BioRender, agreement number CM23SAMGRR.

Cancer is a disease of the genome and with the unprecedented resolution afforded by high‐depth whole‐genome sequencing (WGS) we have discovered a repertoire of cancer‐causing mutations, including single nucleotide variants (SNVs), copy number aberrations (CNAs), small insertions and deletions (termed indels) and structural variants (SVs). While most of these mutations can be classified as ‘innocent’, or unaffecting, passenger events, the acquisition of somatic driver mutations confers a selective advantage in some tumour cells enabling them to clonally expand and outcompete their neighbours [7]. Cancers subsequently develop through Darwinian evolutionary processes in which complete selective sweeps result in an entire population of clonally related cells [8]. The cell of origin that prompted the last complete clonal sweep is called the most recent common ancestor (MRCA) from which all other cancer cells existing in a tumour are descendants. Later in tumour evolution, additional driver mutations may result in incomplete or locally complete clonal expansions [9]. As a result, tumours often contain several subclones that harbour unique subsets of mutations frequently conferring distinctive phenotypic features, such as treatment resistance and the ability to metastasise [10, 11] **(**Figure 1 **).** Table 1 lists the key concepts in tumour evolution.

**Table 1:** Key concepts in tumour evolution.

Concept	Definition
Branching tumour evolution	Tumour clones diverge from the MRCA and evolve in parallel resulting in multiple clonal lineages.
Clone	A lineage of cells descended from the MRCA that inherited the genotype of the MRCA.
Linear tumour evolution	A linear, stepwise accumulation of driver mutations instigating selective sweeps.
Most recent common ancestor (MRCA)	The MRCA is the most recent cell that spawned a set of cells and is a term often used to refer to the genotype of that ancestor cell.
Punctuated tumour evolution	Many genomic aberrations are acquired in a short time burst, often at the earliest stages of tumour evolution.
Subclone	A clone that is a descendant of the MRCA, but has developed additional genomic alterations. These mutations are only present in a subset of tumour cells.

With the advent of massively parallel sequencing, it has become possible to comprehensively catalogue virtually every clonal somatic mutation in a given cancer sample [6]. Most genomic studies rely on DNA sequencing data extracted from millions of cells, with the resulting signals containing intermixed mutations from different tumour subclones, as well as normal cells. Although these methods can provide important information regarding distinct tumour subclones [10, 12], inferring rare populations that could lead to disease relapse or therapeutic resistance remains challenging. This intratumour heterogeneity (ITH) can have adverse consequences for the patient. For example, tumours with complex subclonal structures are often more aggressive, and more likely to metastasise due to an increased ability to adapt to local or systemic factors [13, 14, 15].

Single‐cell sequencing provides a unique opportunity to study ITH and tumour evolution in greater detail when compared to bulk sequencing, often facilitating earlier MRCA inferences. In addition, single‐cell DNA sequencing complements a key role carried out by the diagnostic pathologist for almost 100 years, i.e. the nuclear grading of tumours. On an almost daily basis pathologists use several nuclear grading systems for tumours, such as the Nottingham criteria for breast cancer [16] and the WHO/ISUP grading criteria for renal cell carcinoma [17, 18]. For decades, these nuclear grading systems have been used for prognostication and potentially act as surrogate markers for aberrant genomic processes in tumour cells, most likely including chromosomal aneuploidies, whole‐genome doubling events, or additional large‐scale, complex SVs [19, 20]. As these nuclear phenotypic changes are strongly correlated with tumour aggression and poor patient survival [21, 22], single‐cell sequencing represents a unique opportunity to probe the mutational processes underlying nuclear pleomorphism in cancer in greater detail [21, 22].

There are many excellent reviews of single‐cell transcriptomics primarily aimed at deciphering the tumour microenvironment and ITH [23, 24, 25]. Single‐cell genome profiling is less well explored. In this review, we describe current single‐cell genome sequencing technologies used to investigate clonal dynamics and infer the evolutionary histories of cancer, providing insights into their application and how they can be leveraged to disentangle ITH. We also discuss opportunities to explore ITH in a spatial context using *in situ* sequencing technologies.

## Single cells, ITH, and tumour evolution

Pathologists had long observed that invasive cancers within the colon or breast were likely to develop from premalignant lesions, such as adenomatous polyps with dysplasia or ductal carcinoma *in situ* (DCIS), respectively. First, a step‐wise accumulation of genetic alterations was established in colorectal cancer (CRC) by analysing individual lesions at different histological stages of progression [4, 5], as well as applying micro‐allelotyping techniques in both adenomatous polyps and invasive CRCs [26]. Not only did these studies confirm the order of acquired mutations necessary for CRC development, but they also highlighted substantial allelic heterogeneity between different tissue regions of the same tumour. Similarly, analysis of mutations in known CRC‐causing genes revealed that several distinct subpopulations coexist within both benign adenomatous polyps and invasive CRCs [27, 28]. Heterogeneous patterns of allelic losses were also observed in synchronous DCIS and invasive breast carcinomas, confirming the occurrence of genetic divergence during the clonal evolution of breast cancer [29]. Later, studies combining more advanced molecular genetic analyses in breast cancer, such as HER2 fluorescence *in situ* hybridisation (FISH) and comparative genomic hybridisation (CGH) strategies to explore subclonal chromosomal gains and losses also reported substantial ITH [30, 31, 32, 33]. The biological and clinical context for studying genetic tumour heterogeneity and evolution from bulk sequencing data have been reviewed elsewhere [34, 35, 36].

**Figure 2:**
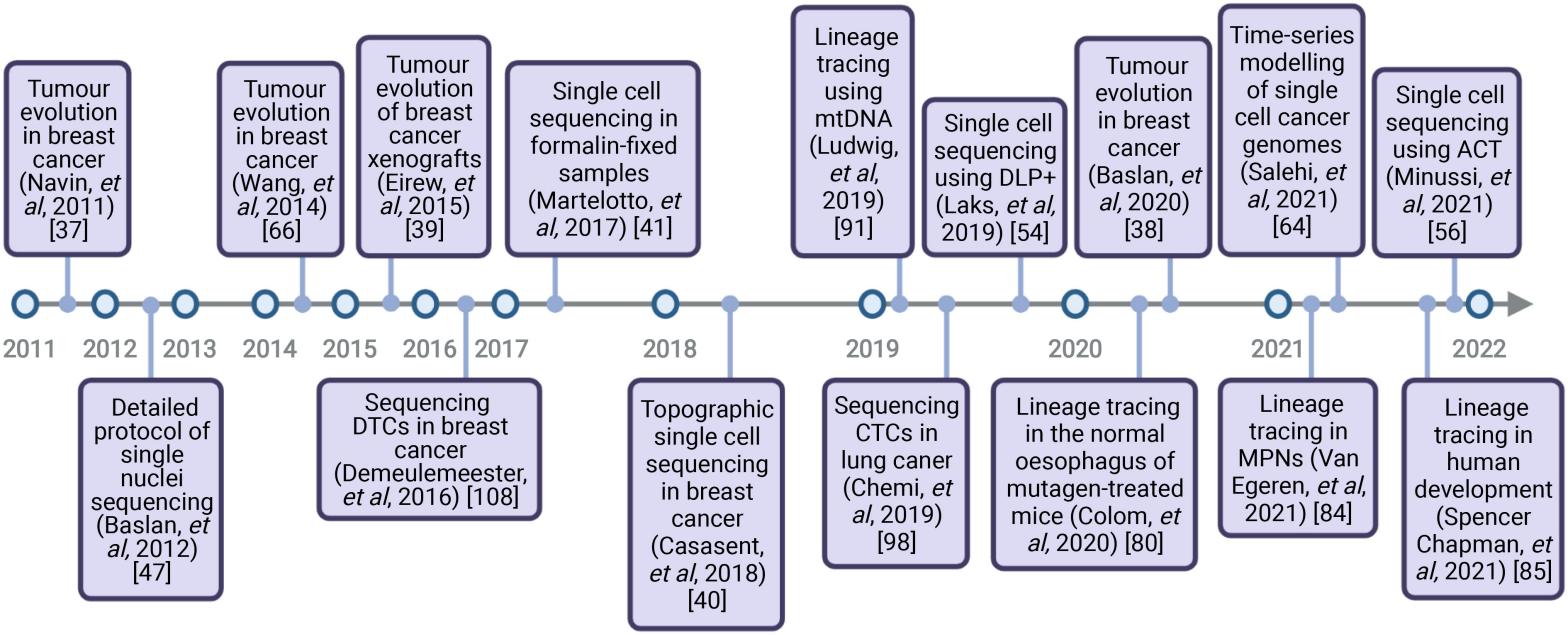
A timeline of single‐cell DNA sequencing studies profiling ITH and tumour evolution. Acoustic cell tagmentation (ACT), colorectal cancer (CRC), circulating tumour cells (CTCs), disseminated tumour cells (DTCs), direct library preparation (DLP), mitochondrial DNA (mtDNA), and myeloproliferative neoplasms (MPNs). Figure prepared with BioRender, agreement number LS23SAQ0V3.

Many recent advances in single‐cell sequencing technologies build on this earlier work, providing the ability to examine the subclonal architecture and evolutionary dynamics of human cancers at a significantly enhanced resolution **(**Figure 2 **).** For example, WGS of single tumour cells in two triple‐negative (ER‐, PR‐, HER2‐) breast cancers revealed the existence of distinct, highly aneuploid, subclonal tumour cell populations that shared partially related but divergent copy number profiles [37]. In one case, a single clonal expansion from the primary breast cancer had seeded the patient's liver metastasis with surprisingly little further copy number evolution. Paradoxically, in both primary breast cancer patients a subpopulation of genetically diverse pseudodiploid tumour cells was also identified that did not appear to travel to metastatic sites. Taken as a whole, these results suggest that tumours grow by iterative clonal expansions, which is entirely consistent with Peter Nowell's theory of cancer evolution published almost four decades ago [9]. Additional single‐cell DNA sequencing studies also demonstrated significant heterogeneity between large‐scale CNAs in primary breast cancers with some subclonal CNAs occurring in genes known to be associated with metastasis, as well as altered therapeutic responses [38, 39].

Building on this work, topographic single‐cell sequencing enables the preservation of important spatial information *via* a combination of laser capture microdissection (LCM) and WGS of isolated single tumour cells [40]. When applied to synchronous DCIS and invasive breast carcinomas, it became apparent that the necessary genomic changes needed for invasion had taken place in ductal units before single aberrant cells had even escaped the confinement of their basement membrane. Intriguingly, single‐cell DNA sequencing revealed that one or more subclonal populations had comigrated through the basement membrane to establish the primary tumour, which also mirrored the results of two previous studies investigating multiclonal invasion in primary breast cancers [41, 42]. Thus, the somatic evolution theory of cancer has been upheld and expanded by multiple single‐cell DNA sequencing studies that have greatly improved our understanding of tumour biology.

## Overview of single‐cell isolation and sequencing strategies

**Figure 3:**
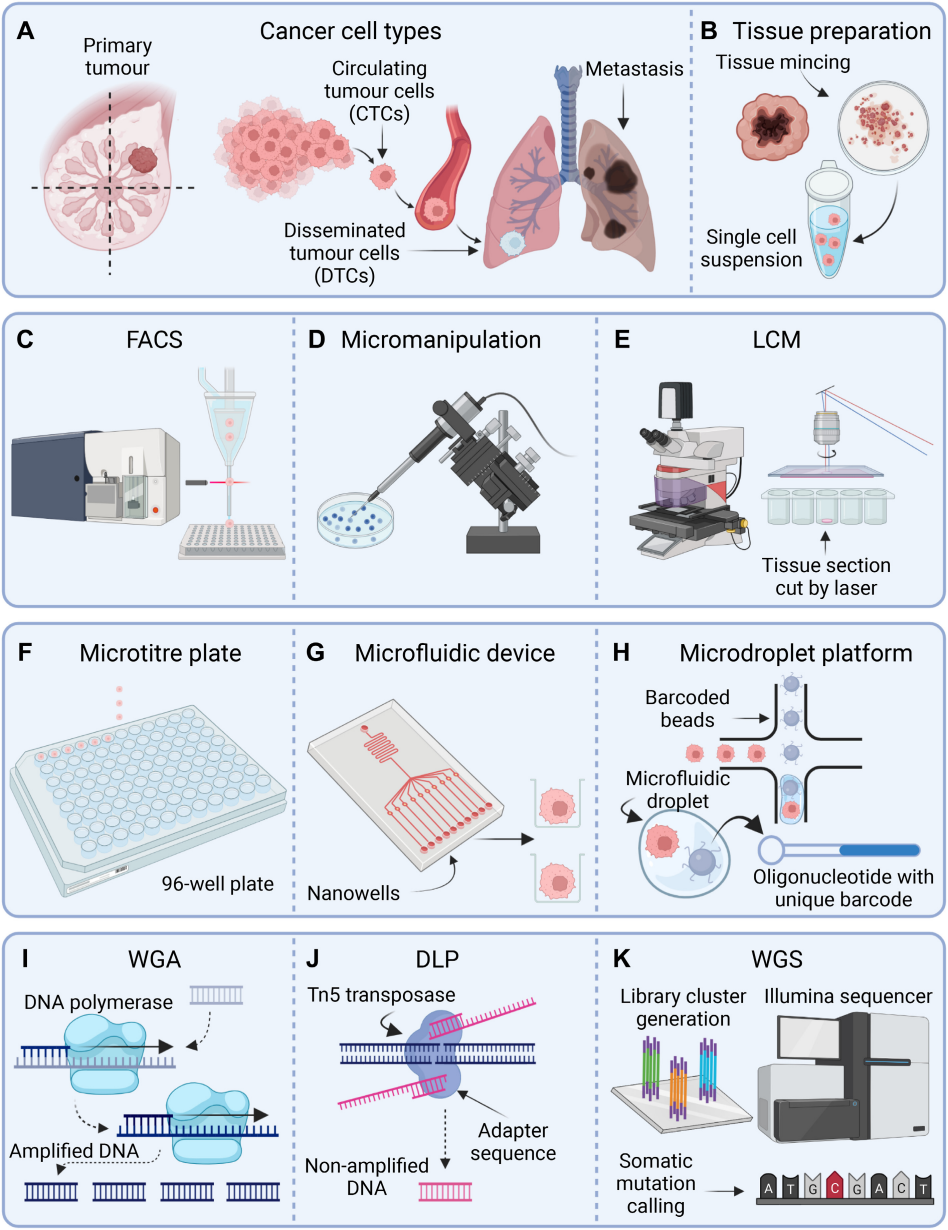
A summary of single‐cell isolation and sequencing strategies. (A) Single‐cell DNA sequencing can be performed on multiple cell types, including single cells isolated from the primary tumour or metastatic lesions, as well as circulating tumour cells (CTCs) and disseminated tumour cells (DTCs). (B) For some single‐cell isolation methods, the tumour will first need to be dissociated to produce a single‐cell suspension. (C–E). Three common methods used for isolating single cells include fluorescent activated cell sorting (FACS), micromanipulation, and laser capture microdissection (LCM). Only LCM techniques can preserve spatial information. (F–H) When working with single‐cell solutions, cells are often isolated into individual reaction chambers prior to single‐cell sequencing, including microtiter plates, nano‐wells in microfluidic devices, or microdroplets. (I) In many workflows, DNA amplification using DNA polymerases is required prior to single‐cell DNA sequencing. (J) Direct library preparation (DLP) techniques use a Tn5 transposase enzyme that simultaneously fragments and ligates synthetic oligonucleotides to single‐cell DNA without the need for preamplification. These oligonucleotides contain sequencing adapters and indexing barcodes. (K) Indexed single‐cell genomic libraries are pooled and sent for WGS, often using Illumina‐based sequencing platforms that use library cluster generation techniques. Following sequencing, genetic mutations are identified. Figure prepared with BioRender, agreement number JK23SAS0N2.

It should be stressed that obtaining high‐quality, single‐cell sequencing data is dependent upon overcoming several technical challenges, including proficient single‐cell isolation, successful DNA extraction (plus or minus DNA amplification), efficacious DNA sequencing, and careful interpretation of the sequencing results, considering any technical biases that may have arisen during the first three steps. In the next section, we will briefly discuss different experimental strategies that can be employed to isolate and sequence single tumour cells **(**Figure 3 **)**.

Isolating single cells from tissue samples with good quality nucleic acids is dependent on appropriate tissue sampling and storage methods. It is critical that tissue dissociation protocols are optimised for tissues of interest to generate high‐quality single‐cell suspensions [43, 44, 45]. The more commonly used methods to isolate single cells include fluorescence‐activated cell sorting (FACS), or micromanipulation either *via* manual (i.e. mouth‐pipetting and the CellTram apparatus) or semiautomated techniques (i.e. di‐electrophoretic DEParray system or CellSearch for circulating tumour cell [CTC] isolation) [45]. Dependent on the research question, single‐cell isolation and FACS using specific phenotypic markers for cells of interest should ideally be performed on fresh tissues [46]. However, in translational research settings this is often not possible due to logistical challenges in obtaining fresh tumour samples. A reasonable compromise is the isolation of single nuclei suspensions from frozen tissues [37, 47]. Once the cell population of interest has been successfully identified, single cells are then captured into individual reaction chambers, such as oil droplets, nano‐wells, or plates [44, 45, 46]. It is important to emphasise that the method for isolating single cells determines the throughput of the experiment (i.e. how many cells), as well as determining the phenotypic information captured. In addition, digestion of solid tissues for single cells comes at a cost of losing spatial information, which could obscure the relationships between cell transcription outputs, as well as interactions between neighbouring tumour cells and the tumour microenvironment. The advantages and disadvantages of different single‐cell isolation strategies are summarised in Table 2.

**Table 2:** The advantages and disadvantages of single‐cell isolation strategies.

Cell isolation method	Description	Advantages	Disadvantages	Examples
**Microtiter‐plate based methods**	Individual cells are deposited into wells within a plate.	Information regarding a cell's phenotype can be collected simultaneously *via* FACS. Individual microscopic images can be recorded enabling the detection of doublets and damaged cells, as well as those with specific morphological features.	Spatial information is lost when tissues are dissociated into a single‐cell suspension. Low throughput method.	Suarez‐Quian *et al* [48] Vermeulen *et al* [49] Leelatian *et al* [43] Casasent *et al* [40]
**Microfluidic array methods**	Microfluidic chips facilitate the isolation of single cells into flow channels, as well as performing library preparation reactions simultaneously.	Single cells can be isolated from small volumes. Microfluidic platforms offer a high single‐cell throughput.	Often requires a uniform cell size. Not appropriate for the analysis of rare cell populations as 90% of cells captured are lost during the experimental process.	Hsiao *et al* [50] Altomare *et al* [51] Yu *et al* [52] Zahn *et al* [53] Tian *et al* [54] Laks *et al* [55]
**Microfluidic droplet‐based methods**	Encapsulates single cells inside a nano‐sized partition that encompasses an oil droplet together with an enzyme coated bead. The bead is loaded with enzymes, adapters and barcodes required to amplify DNA and construct single‐cell sequencing libraries.	Offer the highest throughput for single‐cell experiments. DNA from all single cells can be pooled and sequenced together on the same sequencing run due to unique barcodes. FACS can be used upstream to enrich for a cell population of interest.	Thousands of single cells are sequenced in a single run sometimes leading to insufficient genome coverage. Cell morphology, including information regarding nuclear size is lost.	Nishikawa *et al* [56] Szulwach *et al* [57] Cottinet *et al* [58] Keller *et al* [59]
**Laser capture micro‐dissection (LCM)**	Applies a laser to tissue sections to isolate single cells of interest under direct microscopic visualisation.	LCM methods capture single cells whilst recording their spatial topography. LCM can enrich experiments for single‐cells displaying a specific morphology.	Low throughput method.	Casasent *et al* [40]
**Micro‐manipulation**	The isolation single cells in a solution under direct microscopic visualisation using glass mouth pipettes or robotic micromanipulators (i.e. CellTram). Automated methods such as CellSearch are also available.	Micromanipulation methods can enrich experiments for single cells displaying a specific morphology or phenotype using fluorescent markers.	Low throughput method. Technically challenging. Spatial information is lost when tissues are dissociated into a single‐cell suspension.	Cristofanilli *et al* [60] de Bono *et al* [61] Navin *et al* [37] Demeulemeester *et al* [62]

A significant challenge when working with single‐cell DNA is the limited yield of nucleic acid that is obtained. Each human diploid cell only contains approximately 7 pg of genomic DNA, which until recently has been below the limits of effective library preparation [63]. This can be overcome with whole‐genome amplification (WGA), but this procedure results in several biases and technical artefacts, including a nonuniform genome coverage, as well as allelic imbalances or allelic dropouts, whereby a particular allele is preferentially amplified or not amplified at all, respectively [64, 65, 66, 67, 68]. More recently, a direct library preparation (DLP) method has been introduced whereby indexed genomic libraries are constructed directly from single‐cell DNA without the need for preamplification [53, 55]. In short, DLP techniques use a hyperactive variant of the Tn5 transposase enzyme to simultaneously fragment double‐stranded DNA and ligate synthetic oligonucleotides, including sequencing adapters and single‐cell barcodes. Barcoded genomic libraries can then be pooled for multiplex sequencing. DLP techniques offer substantial gains over traditional WGA methods, including greater coverage uniformity, improved mappability rates, reduced duplicate reads, and a more reliable detection of single‐cell copy number profiles. The accurate measurement of single‐cell genotypes using DLP combined with high‐resolution microscopy images of individual cells, a method referred to as DLP+, has also enabled scientists to decipher the mutational landscape of single tumour cells, whilst simultaneously recording their morphological features, including nuclear characteristics [55]. For DLP and DLP+ techniques, single cells are isolated *via* custom‐made microfluidics devices prior to performing direct tagmentation [55]. However, it is also possible to index individual nuclei that have been FACS‐sorted into plates using well‐specific primers. Although this approach does not require any specialised equipment and uses off‐the‐shelf tagmentation reagents, its throughput is limited due to each cell requiring its own polymerase chain reaction (PCR) reaction [53]. A technique called acoustic cell tagmentation (ACT) also uses Tn5 tagmentation of FACS‐sorted nuclei, which is combined with acoustic liquid transfer technology to perform single‐cell DNA sequencing at a single‐molecule resolution [69]. Moreover, Tn5 tagmentation methodologies have also been applied on intact tissue sections to perform spatially resolved single‐cell DNA sequencing [70]. These novel, nonamplification, Tn5 tagmentation single‐cell DNA sequencing technologies are summarised in Table 3.

**Table 3:** Summary of nonamplification, Tn5 tagmentation single‐cell DNA sequencing technologies.

Nonamplification DNA sequencing technologies	Description	References
Acoustic cell tagmentation (ACT)	Combines FACS of single nuclei, Tn5 tagmentation and acoustic liquid transfer technology to perform high throughput single‐cell DNA sequencing.	Minussi *et al* [69]
DLP	Uses Tn5 transposase enzymes to simultaneously fragment DNA and ligate synthetic oligonucleotides (adapters and index barcodes) to produce genomic libraries without the need for preamplification.	Zahn *et al* [53]
DLP+	As above (DLP) with the addition of high‐resolution microscopy images of individual cells.	Laks *et al* [55]
Slide‐DNA‐Seq	A method that uses barcoded bead arrays combined with Tn5 tagmentation to perform spatially resolved DNA sequencing on intact tissue sections.	Zhao *et al* [70]

## Reconstructing tumour phylogenies at the single‐cell level to investigate clonal dynamics and ITH

Unlike single‐cell transcriptomics, the analytical tools available for single‐cell genomes are less mature. SNV calling is challenging and hugely dependent on the upstream amplification method [64, 65, 66, 67, 71]. More amenable is single‐cell copy number profiling using WGS, which can range from 0.1× to 20× or more in sequencing coverage. Where the identification of large‐scale CNAs (>10 kb) is possible, the phylogenetic history of a tumour can be inferred. Briefly, for single‐cell copy number analysis, mapped reads are assigned to a chromosome position and normalised for GC biases as well as additional amplification artefacts [37, 72, 73]. The relative read depth or number of sequencing reads aligning in each genomic portion are then segmented into regions of consistent copy number states. Integer copy numbers are then assigned to each genomic segment, after which single cells with similar copy number profiles can be grouped into clusters or placed onto a hypothetical evolutionary tree to infer phylogenetic relationships. While hierarchical clustering approaches based on either chromosomal breakpoints [55] or copy numbers can be used to build a tree [69], better phylogenetic models based on parsimony [74] or fully Bayesian interpretation of the data are being actively developed.

Technological advances in single‐cell sequencing and computational workflows now permit the detection of additional mutation types outside of CNAs at a much higher resolution, including SNVs and SVs, facilitating new insights into tumour evolution [69, 75, 76, 77]. However, for phylogeny reconstruction, the combination of matching bulk and single‐cell DNA sequencing data is still recommended, as this enables the interpretation of genomic aberrations at a single‐cell resolution, whilst still maintaining high‐quality mutation calls from the bulk [78].

In theory, library preparations avoiding the biases of amplification allow for a pseudo‐bulk to be obtained from pooled single‐cell reads, reducing sequencing costs. In a recent study, single‐cell DNA sequencing using DLP+ was used to measure clonal genotypes in ~50,000 cells derived from cell lines, xenografts, and diagnostic samples [55]. By aggregating single cells that share similar copy number profiles, clonal genotypes were calculated at a single nucleotide resolution, facilitating key phylogenetic inferences. In addition, analysis of matched genomic and single‐cell imaging measurements revealed correlations between cellular morphology and genome ploidy states, as well as clone‐specific chromosomal aneuploidies. In a separate study also using DLP+ methodologies, CNA‐defined clonal fitness dynamics were determined over time in single tumour cells [77]. Using an hTERT immortalised breast epithelial cell line, the authors demonstrated that *TP53* mutations distributed tumour cell fitness across a larger number of clones with distinct CNAs when compared to wildtype cells. Cisplatin treatment also triggered CNA diversification in primary triple‐negative breast cancer patient‐derived xenografts, leading to the emergence of drug‐resistant clones in previously defined low‐fitness tumour cells. Together, these results suggest that cancer clonal fitness is strongly correlated with CNA accumulation, the dynamics of which may influence the emergence of treatment‐resistant subpopulations. It is also thought that the gradual accumulation of SNVs in tumour cells facilitate extensive clonal diversity and the divergence of specific tumour cell phenotypes, enabling single cells to survive selective pressures such as those induced by the immune system, hypoxia, or chemotherapy [79]. For example, *via* the use of a high‐coverage, single‐cell WGS technology called nuc‐seq, aneuploid rearrangements were found to occur relatively early in tumour evolution, remaining highly stable throughout the clonal expansion of two primary breast carcinomas (one ER+ and the other triple‐negative) [79]. In contrast, point mutations accumulated gradually in breast cancer cells, but led to widespread subclonal diversity. Thus, single‐cell DNA sequencing is an excellent tool for studying somatic diversification in late tumour evolution.

While the importance of studying cancer in an evolutionary framework is now well recognised, relatively little is known about the spatial organisation of cancer subclones and how they might influence tumour growth and metastasis. Recently, the development of slide‐DNA‐seq, a method that captures spatially resolved DNA sequences from intact tissue sections at an almost single‐cell resolution, has enabled scientists to better decipher the role of spatial genomics in cancer progression [70]. The authors applied slide‐DNA‐seq to a genetically engineered mouse model of lung adenocarcinoma and a primary human CRC, demonstrating that clonal populations are indeed confined to specific spatial regions with clone‐specific transcriptomic changes reflecting divergent evolutionary paths. In addition, a relatively new single‐cell RNA‐based methodology called Base Specific *In Situ* Sequencing (BaSISS) was used to generate quantitative, histology‐based maps of cancer subclones in two multifocal breast cancers [80]. Here, the authors found that coexistent genetic clones had distinct transcriptional, histological and immunological characteristics and that the patterns of spatial genetic heterogeneity were strongly influenced by resident tissue structures. Additional *in situ* mutation technologies such as Basescope are also in existence, which uses RNA *in situ* hybridisation to detect point mutations in tissue sections, providing valuable insight into the emergence of subclones in human cancers [81]. Thus, as spatially resolved DNA and RNA sequencing is pushed to the single‐cell level, these technologies will provide new opportunities to determine how cell‐intrinsic and cell‐extrinsic factors contribute to ITH and tumour evolution.

## Leveraging single‐cell sequencing to perform lineage tracing in normal and malignant tissues

Recent studies of somatic mutations in normal human tissues have illuminated how preneoplastic and early cancer genomes are potentially shaped by a life‐long process of mutational accumulation that occurs with normal ageing [82, 83, 84, 85, 86, 87]. Simultaneously, innovations in single‐cell genomics have permitted detailed investigations of the lineage commitment and differentiation of preneoplastic cells, improving our understanding of how cellular dynamics influence ageing and cancer development. In model organisms, most cell lineage approaches rely on engineered genetic labels tagging single cells with heritable markers, such as fluorescent reporter genes, Cre‐mediated recombination, or CRISPR‐based genetic scars [88, 89, 90, 91, 92]. Combining these tracing methods with single‐cell sequencing approaches has enabled scientists to interrogate both lineage fates and phenotypes of single cells simultaneously. By performing transgenic lineage tracing in mutagen‐treated mice, deep sequencing of the normal oesophageal surface epithelial revealed strong clonal competition over time, with multiple genes under positive selection, including *Trp53*, *Notch1*, and *Notch2* [93]. Intriguingly, when a mutant clone collides with another of a similar competitive fitness, mutant clones tended to revert to a homeostatic fate, suggesting a constraining neighbour‐regulated fitness programme for cancer prevention in the normal, ageing oesophagus.

As the genetic manipulations required for lineage tracing can only be applied to animal or *in vitro* cancer models, lineage tracing in humans is restricted to retrospective approaches, including the detection of somatic mutations [94]. For example, the pathognomonic JAK2‐V617F mutation underlies the manifestation of myeloproliferative neoplasms [95, 96]. Therefore, lineage tracing of somatic mutation patterns in the *JAK2* gene of haematopoietic stems cells (HSC) has enabled scientists to identify when this mutation first occurred and how it influences cell behaviour [97]. Astonishingly, JAK2‐V617F mutations were found to occur in a single HSC several decades prior to a malignant diagnosis in treatment‐naive patients, inducing selective advantages in HSCs. In line with these findings, lineage tracing of single‐cell‐derived haematopoietic colonies in healthy foetuses has shown that haematopoietic progenitors already acquire tens of somatic mutations by 18 weeks of gestation [98]. Similarly, WGS of ~1,000 clonal haematopoietic colonies from patients with myeloproliferative neoplasms demonstrated that the acquisition of JAK2‐V617F driver mutations often occurred early in life, including during the *in utero* period [99].

Taken further, lineage tracing using WGS and somatic mutation calling in normal tissues has the potential to transform our understanding of somatic mutagenesis, ageing, and cancer development. In one study using NanoSeq or nanorate sequencing, a duplex approach that sequences both strands of a DNA molecule to reduce sequencing errors, differentiated cells in the blood and colon displayed remarkably similar mutational loads when compared to their corresponding stem cells, despite blood cells having undergone considerably more cell divisions [100]. Similarly, postmitotic neurons were also found to have accumulated somatic mutations at a similar rate to those of mitotically active tissues despite, the absence of cell division. Lineage tracing has also been applied to investigate nongenetic drivers of cancer therapy failure and the acquisition of a drug‐tolerant ‘persister’ state in some tumour cells [101]. For example, although most persister cells remain arrested in the presence of a drug, some may reenter the cell cycle despite continued treatment [102]. The application of Watermelon, a high‐complexity expressed barcode lentiviral library for simultaneous tracing of clonal lineages, as well as proliferation and transcriptional states in single tumour cells, showed that persister cell proliferative capacity is associated with antioxidant gene upregulation and a shift to fatty acid oxidative metabolism, exposing new vulnerabilities in tumour cells that may be amenable to therapeutic intervention [101].

As technologies aiming to detect somatic mutations by WGS in single cells are still in their infancy and had previously been difficult to apply at scale, lineage‐tracing studies using mitochondrial DNA (mtDNA) sequence variants have also been successfully applied to infer clonal relationships in normal tissues. In one study, lineage tracing of mtDNA mutations was used to observe the clonal evolutionary dynamics of stem cell populations within the human colon [103]. Recently, single‐cell RNA and ATAC sequencing has also been used to track somatic mutations in mtDNA, providing the ability to relate clonal cell dynamics to gene expression and chromatin accessibility [104]. In addition, a new single‐cell transposon‐based WGA method, termed multiplexed end‐tagging amplification of complementary strands (META‐CS), has significantly improved single‐cell SNV detection rates [105]. This technology takes advantage of the complementary strands of double‐stranded DNA to filter out false‐positives and achieves a high accuracy for detecting SNVs in single cells.

## Single‐cell sequencing, CTCs, and disseminated tumour cells

The isolation and analysis of CTCs from peripheral blood represents an alternative strategy to investigate tumour progression and evolution [106]. In the last decade, a wealth of studies has successfully enumerated the clinical relevance of studying CTC single‐cell genomes, which have been comprehensively reviewed [106, 107, 108]. CTC status is known to be a prognostic marker in multiple human cancers, including nonsmall‐cell lung cancer (NSCLC) and CRC [109, 110]. For example, pulmonary venous CTCs were successfully detected in 48% of 100 NSCLC patients in the TRACERX study (ClinicalTrials.gov NCT01888601), which was significantly correlated with lung‐cancer‐specific relapse [111]. In a single genomic case study of one NSCLC patient, genomic profiling of pulmonary venous CTCs revealed a 91% SNV mutational overlap with a metastatic lesion detected at 10 months when compared with a 79% SNV mutational overlap in the primary tumour. Although most CTCs are single cells, a small proportion circulate as a group of clustered cells comprising two or more nuclei [112]. CTC clustering is thought to be part of an adaptive mechanism that improves CTC survival and enhances their metastatic potential, as CTC clusters have been shown to metastasise up to 100 times more effectively when compared to their single‐cell counterparts [113, 114, 115]. As CTCs and CTC clusters are thought to drive metastasis formation in many human cancers, the demand for therapies that specifically target these single or clustered tumour cells is extremely high. Thus, the analysis of CTCs and CTC clusters at a single‐cell resolution could offer a unique, minimally invasive approach to characterise and monitor dynamic changes in ITH in cancer patients, as well as providing future avenues for therapeutic exploration.

CTCs can extravasate and inhabit distant organs, where they are referred to as disseminated tumour cells (DTCs), which may remain dormant for many years or provide a reservoir of malignant progenitor cells for distant metastases [116, 117]. There is increasing evidence that primary tumours have systemic effects beyond seeding of DTCs; for example, *via* immunosuppression or extracellular matrix remodelling [118]. Therefore, DTCs could benefit in their niches from primary tumour‐induced effects, which may be essential for early DTC colonisation [119]. Intriguingly, DTCs isolated prior to metastasis often lack the characteristic genomic alterations typical of the metastatic tumour, suggesting that DTCs need to acquire additional genomic alterations prior to metastasis formation or that they could help to prepare the metastatic niche for late‐arriving DTCs [120]. In contrast, late DTCs may remain dormant until unknown signals stimulate reentry into the cell cycle and take over the formation of metastases. Importantly, the role of early, genomically immature DTCs in metastasis and tumour progression is still under intense debate. For example, tumour‐specific truncal mutations were completely absent in the subset of DTCs isolated from the bone marrow of nonmetastatic breast cancer patients, suggesting that they did not arise from the MRCA [62]. Thus, the detailed genomic profiling of single DTCs is still required to decipher the exact roles of early and late disseminating cancer cells in metastasis formation.

## Correlating genotype with phenotype in single‐ cells

Single‐cell RNA sequencing is a well‐established, fundamental tool for characterising cell phenotypes, reviewed elsewhere [121, 122, 123]. Like single‐cell DNA sequencing technologies, low‐throughput, plate‐based methods (i.e. Smart‐seq2 [124] and CEL‐Seq2 [125]), as well as high‐throughput droplet [126, 127] or nano‐well‐based techniques [128, 129] are in existence, whereby specific barcodes in individual reaction chambers detect all the complementary DNA sequences generated from a single cell. The rapid growth in the scale and robustness of single‐cell RNA sequencing protocols has paved the way to substantial scientific discoveries, including the commencement of an extensive international initiative called The Human Cell Atlas, aiming to build comprehensive RNA reference maps for all human cell types [130]. To understand the molecular processes leading to cell phenotypic differentiation more comprehensively, new technologies are required that simultaneously assay different types of molecules in the same cell (i.e. DNA and RNA). Such multi‐omics approaches would offer a direct link between a cell's modified genome and its transcriptomic landscape. In addition, cell lineage trees generated from single‐cell WGS data can be phenotypically annotated reflecting the types and states of cells that are present.

In an approach termed G&T‐seq (genome and transcriptome sequencing), a single cell's polyadenylated (polyA) RNA can be separated from its genomic DNA using a biotinylated oligo‐dT primer [131]. Thereafter, both a single‐cell's genome and transcriptome are amplified in parallel followed by sequencing. This integrated method can be used to expose the diverse effects of genetic variation on transcript levels [131]. Additional single‐cell multi‐omic protocols aiming to sequence both the cell's DNA and RNA simultaneously have also been devised, including DR‐Seq and direct nuclear tagmentation and RNA sequencing (DNTR‐seq), both of which were able to link single‐cell states to corresponding genetic alterations, enabling routine analysis of heterogenous tumours and additional complex tissues [132, 133].

The tumour microenvironment and nongenetic adaptive mechanisms also impact cell phenotypes and tumour progression [134]. New technologies such as *Visium* [135], Nanostring DSP [136], and slide‐RNA‐seq [137] permit spatially resolved transcriptomics at a single‐cell or near single‐cell resolution, capturing *in situ* RNA sequences from intact tissue sections. These techniques enable the accurate spatial analysis of gene expression, the results of which can be easily integrated with large‐scale single‐cell RNA sequencing datasets to facilitate cell phenotype examination in both normal and malignant tissues. It is hoped that future advances in spatially resolved single‐cell RNA sequencing will provide new opportunities to explore ITH and tumour evolution, whilst considering the spatial locations of both normal and tumour cells.

## Conclusions and perspectives

The rate of development of single‐cell sequencing technologies is increasing rapidly, providing the ability to resolve tumour phylogenies in unprecedented detail with less uncertainty when compared to bulk sequencing data. Further progress is likely to be achieved by refining existing single‐cell sequencing techniques to improve their sensitivity and accuracy. As discussed above, nonamplification, Tn5 tagmentation single‐cell DNA sequencing methodologies, such as DLP, DLP+, and ACT, are likely to replace older methods relying on the amplification of genomic DNA by PCR, thereby removing unwanted technical artefacts that could lead to false mutation calls. In addition, technological advances in microfluidic droplet and microfluidic array‐based platforms will facilitate an increasingly high single‐cell throughput, processing thousands of single cells in a single experimental run.

Linking cell morphology with genomic changes will become more frequent *via* the use of live cell imaging prior to single‐cell sequencing. In addition, new technologies, such as slide‐DNA‐seq and slide‐RNA‐seq, will continue to improve in efficacy and resolution, permitting spatially resolved analyses, whilst preserving tissue architecture. Likewise, alternative methods that facilitate multiple omic layers, such as joint genomics and transcriptomics, are likely to increase in use, permitting the simultaneous assessment of both cell genotype and phenotype.

For accurate tumour evolution inference, the combination of matching bulk and single‐cell DNA sequencing data is still recommended, as this enables a greater ability to resolve phylogenetic relationships. However, as single‐cell DNA sequencing technologies improve their resolution, this may no longer be necessary in the future. Lastly, novel computational methods that permit ever‐increasing complex analyses of somatic mutation acquisition in single cells, as well as clustering multiple types of somatic mutations between thousands of single cells will be required. It is hoped that the advancement of single‐cell sequencing technologies will greatly improve our understanding of ITH and tumour evolution, leading to novel biological insights and the development of innovative therapies for cancer patients.

## Author Contributions Statement

AB, PVL and NP conceived the concept and designed the article. AB performed the literature search and drafted the article and figures. AB, MT, PVL and NP were responsible for reviewing and editing the article.
